# Transcriptomic signatures differentiating carrier and dead-end fish hosts of proliferative kidney disease

**DOI:** 10.3389/fcimb.2026.1856713

**Published:** 2026-07-02

**Authors:** Gokhlesh Kumar, Arun Sudhagar, Saloni Shivam, Reinhard Ertl, Mona Saleh, Mansour El-Matbouli

**Affiliations:** 1 Marine Biotechnology, Fish Nutrition and Health Division, ICAR-Central Marine Fisheries Research Institute, Kochi, India; 2 Clinical Division of Fish Health, University of Veterinary Medicine, Vienna, Austria; 3 Peninsular Aquatic Genetic Resources Centre, ICAR-National Bureau of Fish Genetic Resources, Kochi, India; 4 Aquatic Environment and Health Management Division, ICAR-Central Institute of Fisheries Education, Rohtak, India; 5 VetCore Facility for Research, University of Veterinary Medicine, Vienna, Austria

**Keywords:** differentially expressed transcripts, Myxozoan parasite, proliferative kidney disease, salmonids, *Tetracapsuloides bryosalmonae*

## Abstract

**Introduction:**

*Tetracapsuloides bryosalmonae* is the causative agent of proliferative kidney disease (PKD) in salmonids. Brown trout function as a carrier host, while rainbow trout serve as a dead-end host. However, the molecular mechanisms underlying these host-specific differences remain poorly understood. Therefore, in the present study, we aimed to identify host-specific transcriptomic signatures distinguishing carrier and dead- end host.

**Method:**

Comparative transcriptomic analysis of infected kidneys from brown trout and rainbow trout was performed to identify differential gene expression patterns associated with host-specific responses to *T. bryosalmonae*. Differentially expressed transcripts unique to each host species were identified and subjected to functional enrichment analyses.

**Results:**

A total of 636 and 1,348 unique transcripts were upregulated and downregulated in the infected kidney of brown trout, respectively. In contrast, 526 and 586 unique transcripts were upregulated and downregulated in the infected kidney of rainbow trout, respectively. The uniquely upregulated transcripts in infected brown trout were mainly associated with cell cycle and mitotic cell cycle–related processes, whereas in infected rainbow trout, they were associated with the regulation of biological quality, intracellular signal transduction, and response to chemical stimulus. Similarly, uniquely downregulated transcripts in infected brown trout were mainly associated with anatomical structure morphogenesis, cellular developmental processes, cell differentiation, animal organ development, and cell surface receptor signaling, whereas in infected rainbow trout, they were associated with transmembrane transport, ion transport, and nervous system development.

**Discussion:**

Our findings suggest that host-specific transcriptomic signatures and their differential regulation underlie the contrasting immune responses of brown trout and rainbow trout against *T. bryosalmonae*. Such distinct transcriptomic regulation facilitates intra-luminal sporogonic stages of the European strain of *T. bryosalmonae* in the kidneys of the carrier host, while restricting its development in the dead-end host. Overall, these findings indicate that brown trout maintains a regulated, proliferation-associated environment compatible with parasite development, whereas rainbow trout mounts an immune-dominated response that may restrict parasite sporogenesis. This study provides new insights into the molecular basis of host-specific outcomes in PKD and highlights the role of transcriptional regulation in shaping host–parasite interactions.

## Introduction

Proliferative kidney disease (PKD) significantly affects both farmed and wild salmonid populations, causes economic losses and endangers wild fish populations. PKD is caused by the endoparasite *Tetracapsuloides bryosalmonae* (PKX cnidarian myxozoan, phylum Cnidaria and class Malacosporea) (Feist et al., 2001). Declining wild trout populations in European and North American rivers have been linked to PKD, which may pose a major threat to freshwater salmonids (Bettge et al., 2009; Philpott et al., 2025; Dash and Vasemägi, 2014). Proliferation of *T. bryosalmonae* in highly susceptible hosts such as Atlantic salmon (*Salmo salar*) and brown trout (*Salmo trutta*) induces a pronounced granulomatous inflammatory response within the interstitial tissues, leading to marked splenomegaly and severe renal swelling. This is accompanied by extensive destruction of renal parenchyma, interstitial oedema, and diffuse infiltration of macrophage- and lymphocyte-like cells, often associated with characteristic PKX cells within the lesions (Clifton-Hadley et al., 1987; Sterud et al., 2007). Similarly, in rainbow trout (*Oncorhynchus mykiss*), the disease is characterized by severe proliferative and granulomatous nephritis, with marked kidney enlargement resulting from lymphoid hyperplasia and extensive leukocyte infiltration. Histopathological alterations include disruption of renal architecture, degeneration of haematopoietic tissue, and compression or loss of renal tubules (Bettge et al., 2009).

At the cellular and molecular levels, PKD is associated with significant dysregulation of B- and T-cell responses in salmonids, contributing to ineffective parasite clearance and disease progression (Gorgoglione et al., 2013; Bailey et al., 2019a; Saura Martinez et al., 2025). Both brown trout and rainbow trout exhibit strong B-cell activation with increased expression of immunoglobulin-related genes during *T. bryosalmonae* infection. In brown trout, parasite-specific antibody production is accompanied by upregulation of secretory IgM (IgM sec) and Blimp1, along with repression of Pax5, suggests differentiation toward antibody-secreting plasma cells (Kumar et al., 2014, 2015; Bailey et al., 2019a; Shivam et al., 2021). In contrast, rainbow trout show elevated IgM sec, Blimp1 and Pax5 expression (Bailey et al., 2017). T-cell responses during PKD are also altered, with brown trout exhibiting upregulation of Th1-associated markers such as Tbet, IFN-γ and TNF-α during advanced stages of infection, whereas studies in rainbow trout suggest a more complex interplay between Th1- and Th2-like responses (Wang et al., 2010; Gorgoglione et al., 2013; Bailey et al., 2017).

*T. bryosalmonae* has a two-host life cycle involving both vertebrate (fish) and invertebrate (bryozoan) hosts. The freshwater bryozoans act as its primary host and serve as reservoirs of *T. bryosalmonae* spores infective to their secondary hosts, the salmonid fish (Morris and Adams, 2006; Kumar et al., 2020). The parasite undergoes a series of developmental changes within both the primary and secondary hosts (McGurk et al., 2006; Morris and Adams, 2008). Spores released from infected bryozoan colonies mainly enter through their gills and migrate to the blood circulatory system and then reach the main target organ, the kidney. Within the kidney, the parasite develops through extrasporogonic and sporogonic stages (Morris and Adams, 2008). Notably, the extrasporogonic stages trigger a pronounced granulomatous inflammatory response, leading to characteristic microscopic lesions in the affected organ (Ellis et al., 1985). During advanced stage of infection, disruption of haematopoietic tissues contributes to anaemia and immunopathology (Hoffmann and Lommel, 1984; Hedrick et al., 1993). Subsequently, an intratubular development of the parasite leads to the formation of the spores, which leave the infected fish via urine and are infective to the freshwater bryozoans (Morris and Adams, 2006). In addition, *in vivo*–induced antigens of *T. bryosalmonae* expressed in brown trout during parasite development have been identified using *in vivo*–induced antigen technology, contributing to the understanding of *T. bryosalmonae* virulence mechanisms in salmonids (Kumar et al., 2022).

European strains of *T. bryosalmonae* spores develop in the kidney tubules of brown trout (*Salmo trutta*) and infected brown trout release viable parasite spores via the urine into the water, where they are transmitted to bryozoans, completing the parasite’s life cycle. Therefore, brown trout are known to be carrier of *T. bryosalmonae* European strains (Morris and Adams, 2006; Soliman et al., 2018). In contrast, rainbow trout exhibit similar clinical manifestations upon infection but do not shed the viable spores needed to infect the bryozoans and cannot complete the parasite’s life cycle. Thus, rainbow trout represents a dead-end fish host (Grabner and El-Matbouli, 2008; Kumar et al., 2013). However, North American strains of *T. bryosalmonae* can form spores in the kidney tubules of rainbow trout (Hedrick et al., 2004), though it was not been proven whether infected rainbow trout transmit the spores to bryozoans. However, a recent study has demonstrated that North American strains of *T. bryosalmonae* can complete transmission between *O. mykiss* and the freshwater bryozoan *Fredericella borealis*, confirming rainbow trout as an active vertebrate host capable of shedding viable infectious spores in North America and emphasizing the epidemiological risk associated with fish stocking and translocation (Das et al., 2026). This led to the hypothesis that there are two lineages of *T. bryosalmonae*: one adapted to the genus *Salmo* and the other to the genus *Oncorhynchus.* This hypothesis was subsequently confirmed by using internal transcribed spacer sequence analyses, which resolved distinct European and North American lineages of *T. bryosalmonae* (Henderson and Okamura, 2004). In addition to differences in parasite development, rainbow trout populations from Europe and North America exhibit distinct host responses to infection. European rainbow trout infected with European strains typically develop severe proliferative kidney disease (PKD), characterized by marked renal swelling, lymphoid hyperplasia, and granulomatous inflammation, while still restricting completion of the parasite life cycle (Grabner and El-Matbouli, 2008; Bailey et al., 2017). In contrast, North American rainbow trout infected with North American strains permit intratubular sporogony and exhibit a comparatively more permissive host–parasite interaction, suggestive of parasite development (Hedrick et al., 2004; Okamura et al., 2011). Furthermore, we identified noticeable differences in the transcriptomic patterns of *T. bryosalmonae* derived from the kidneys of brown trout and rainbow trout (Shivam et al., 2023).

Studies on the global transcriptome profiles of host-parasite interaction of brown trout in comparison to rainbow trout are limited. Earlier, suppression subtractive hybridization was used to compare m-RNA expression of infected kidneys of brown trout and rainbow trout in response to *T. bryosalmonae*, revealing differential expression of anti-inflammatory, cell proliferation and immune-relevant genes (Kumar et al., 2014; 2015). Subsequently, the global transcriptome analysis was performed to investigate the biological insights of transcripts in the kidney of brown trout and rainbow trout at the active phase and late phase of *T. bryosalmonae* using RNA-seq (Sudhagar et al., 2019; Bailey et al., 2019b). Furthermore, differentially expressed alternatively spliced transcripts were identified in the infected kidney of brown trout during PKD, providing insights into alternative splicing events during host–parasite interactions (Sudhagar et al., 2022).

Understanding the dynamic interactions underlying responses in carrier versus dead-end hosts can be achieved, in part, by comparing the transcriptomes of both fish species. It is also important to understand how host species influence parasite development and behavior, particularly given the differences in parasite stage development between these two fish species. RNA-seq analysis can be used to elucidate how the host modulates parasite developmental processes and mechanisms during infection of the target organ. To this end, we aimed to identify unique transcriptomic signatures in the kidneys of brown trout (carrier host) and rainbow trout (dead-end host) that distinguish them during *T. bryosalmonae* development using high-throughput transcriptome sequencing. To elucidate the molecular mechanisms underlying responses in both carrier and dead-end fish hosts, we performed enrichment analysis and functional annotation of the differentially expressed genes (DEGs), involved in a plethora of biological roles in different biological processes and pathways.

## Methods

### Laboratory exposure of brown trout and rainbow trout

Juvenile brown trout and rainbow trout used in this study originated from our previous experiment and were sourced from Glück Fischzucht GmbH, Mauerkirchen, Austria (Kumar et al., 2013). Briefly, prior to the start of the experiment, ten fish from each species were randomly sampled, and their health status was assessed using routine diagnostic procedures. Parasitological examination was done by microscopic analysis of wet mounts from skin mucus, gill squashes, and kidney smears. For bacterial examination samples from kidney and spleen were plated on TSA-blood agar. Viral examination was done using PCR assays targeting VHSV, IPNV and IHNV following standard diagnostic protocols. All examined fish tested negative for parasites as well as bacterial and viral pathogens. Sixty brown trout and 60 rainbow trout (mean length 5.5 ± 0.5 cm, mean weight 2.3± 0.5 g) in three replicates (20 fish per replicate) were exposed to the spores of *T. bryosalmonae* released from the laboratory infected bryozoan *F. sultana* colonies (Kumar et al., 2013). The parasite was maintained through an established laboratory life cycle using *F. sultana* colonies originating from a clonal population collected in Germany (Grabner and El-Matbouli, 2008; Kumar et al., 2013; Saleh et al., 2026). Free *T. bryosalmonae* spores in suspension, released from 12 mature sacs, were added to all aquaria, which were then maintained with vigorous aeration for 24 h at 16.5 ± 1 °C. Following exposure, both fish species were maintained in 100-litre volume of re-circulating water in aquaria at 16.5 ± 1 °C. Additionally, 30 brown trout and 30 rainbow trout were held as un-exposed control groups. Fish were fed *ad libitum* daily with commercial trout feed (Garant Aqua, Austria) according to our established protocol. Posterior kidneys were sampled from both exposed (n = 10) and un-exposed control (n = 5) groups at different time points (6, 8, 10, 12, 14 and 17 wpe) from different replicate tanks for each experimental group. Each tissue was equally sliced on a glass slide by using a sterile scalpel blade to maintain homogeneity and reproducibility, and subsequently divided into two portions. One portion was preserved in RNA-later, while the second portion was fixed in 10% neutral buffered formalin. Tissues preserved in RNA-later were stored at -80 °C for further use.

### Sample selection

The optimal time point (10 wpe) for RNA-seq was determined by the presence of high numbers of intra-luminal sporogonic stages and interstitial pre-sporogonic stages in brown trout and only high numbers of interstitial pre-sporogonic stages in rainbow trout, observed using histological and immuno-histological examination. Based on these findings, we selected kidney samples at 10 wpe for transcriptomic analysis. A description of parasite stages and the transmission process is presented in a flowchart (**Figure 1**).

**Figure 1:**
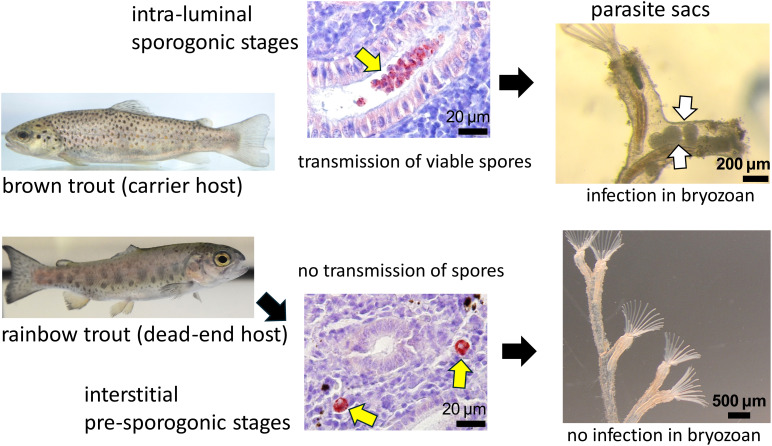
The parasite stages and transmission process of *T. bryosalmonae*. Spores released from infected bryozoans enter fish via the gill epithelium and subsequently develop in the kidney. In brown trout, intraluminal sporogonic stages of the parasite develop within the kidney tubules, whereas in rainbow trout, only interstitial pre-sporogonic stages are observed. Infected brown trout release viable spores into the water via urine and transmit the parasite to bryozoans and complete the life cycle of *T. bryosalmonae*. However, infected rainbow trout lack intraluminal sporogonic stages in the tubules of the kidney and are unable to transmit the parasite to bryozoans and are therefore considered a dead-end host.

### RNA-sequencing

Total RNA was extracted from five independent samples from each exposed and un-exposed control fish species at 10 wpe using the RNeasy mini kit (Qiagen, Germany) including on-column DNase treatment. Integrity of each RNA sample was verified on a 4200 TapeStation system using the RNA ScreenTape Kit (Agilent, USA). Only samples with a RIN value above 8.0 were used for RNA-sequencing (**Supplementary Material 1**).

c-DNA libraries were prepared with 1 µg total RNA input using the Poly(A) RNA Selection Kit V1.5 and the CORALL mRNA-Seq Library Prep Kit (Lexogen, Austria) according to the manufacturer’s protocol. Quality control of each library was checked on the 4200 TapeStation with the D1000 ScreenTape Kit (Agilent, USA). RNA sequencing was carried out on a NovaSeq 6000 system (Illumina, San Diego, USA) implementing 100-bp paired-end reads. The sequencing was performed by the Next Generation Sequencing Facility at Vienna BioCenter Core Facilities, Vienna BioCenter, Austria.

### RNA-seq analysis

The raw sequencing reads were processed for quality filtering with Trimmomatic v0.39 (Bolger et al., 2014) to remove reads shorter than 50 nucleotides, adapter sequences and low-quality bases (Phred score ≤ 30). The clean reads were mapped to the reference genomes of brown trout fSalTru1.1 (GCA_901001165.1) and rainbow trout USDA_OmykA_1.1 (GCA_013265735.3) obtained from Ensembl database (version 115). The mapping of sequencing reads was carried out in OmicsBox 2.2.4 software (BioBam, Spain) using the Burrows-Wheeler Alignment algorithm (Li and Durbin, 2009). The number of detected genes reported by OmicsBox corresponded to 29,179 genes for brown trout and 21,952 genes for rainbow trout from the Ensembl annotations used in the analysis. The generated count tables were used for pairwise differential expression analysis by comparing parasite-exposed samples with the respective controls using the edgeR package in OmicsBox (Robinson et al., 2010). Differentially expressed genes were filtered by FDR *p*-values < 0.01, log2 fold changes < -1 or >1, and a count-per-million (CPM) filter > 1.5, to exclude lowly expressed genes. The background gene list comprised of all expressed genes in the samples, showing a CPM > 1.5. Venn diagrams were generated with Venny (Oliveros, 2015).

### Gene ontology and KEGG pathway analysis

The lists of up- and downregulated genes for both fish species were analyzed for enriched Gene Ontology (GO) terms and KEGG pathways in ShinyGO 0.77 (Ge et al., 2020). The following settings were used: species: best matching species, FDR cut-off: 0.05, pathway size: min. = 3 and max. = 2000, and pathway database: GO Biological Process and KEGG. Zebrafish (*Danio rerio*) was detected as the best matching species for both fish species gene-sets and was kept to maintain the comparability of the results. REVIGO (Supek et al., 2011) was used to reduce the redundancy of the lists of enriched GO terms obtained from ShinyGO, with the following settings: similarity: medium, species, whole UniProt database and semantic similarity measure, SimRel.

### Validation of differentially expressed transcripts

To test the validity of RNA-seq results, we performed a quantitative analysis of six randomly selected differentially expressed transcripts by quantitative real-time PCR (qRT-PCR) using a CFX96 Touch Real-Time PCR detection system (Bio-Rad). The analysed transcripts included apelin receptor 2 (APLNR2), B-cell receptor (CD22), cathepsin-B (CTSB), TSC22 domain family protein 3 (TSC22D3), toll-like receptor 1 (TLR1) and suppressor of cytokine signaling 3 (SOCS3). cDNA was synthesized from total RNA (1 µg) using an iScript cDNA Synthesis Kit (Bio-Rad), according to the manufacturer’s protocol. The cDNA samples (*n* = 5) from exposed and unexposed control fish species groups were subjected to qRT-PCR with three technical replicates using specific and optimized primers (**Supplementary Material 2**).

qRT-PCR reaction was performed in a final volume of 20 μl, which contained 4 μl of cDNA (1:10 diluted), 0.5 μM of each primer, 1X SsoAdvanced™ Universal SYBR Green Supermix (Bio-Rad), and sterile DEPC-treated distilled water. qRT-PCR consisted of an initial denaturation at 95 °C for 5 minutes, followed by 37 cycles of denaturation at 95 °C for 30 seconds, annealing at 55-61 °C for 30 seconds, and elongation at 72 °C. A final elongation step was performed at 95 °C for 30 seconds. A melting-point curve analysis was carried out from 55–95 °C with an increment of 0.5 °C per 10 seconds to verify amplification specificity and detect non-specific PCR products. Elongation factor alpha and beta-actin were used as reference genes to normalize the test samples. The relative gene expression of host genes between exposed and control groups was calculated using the 2^−ΔΔ*C*t^ method. The statistical difference between groups was determined using two-tailed unpaired Student’s *t*-test with Welch’s correction. Linear regression analysis was conducted using the corresponding log_2_ fold-change values obtained from RNA-seq and qRT-PCR to assess the relationship between the two datasets. For all statistical analyses, a *P* value < 0.05 was considered statistically significant.

## Results

### Histological analysis

Moderate renal hypertrophy (grade 2-3) and splenomegaly were observed in both brown trout and rainbow trout at 10 wpe. Furthermore, numerous intraluminal sporogonic and pre-sporogonic stages of the parasite were observed in the kidneys of brown trout (**Figure 2A**), whereas only interstitial pre-sporogonic stages were detected in the kidneys of rainbow trout (**Figure 2B**) at the same time point.

**Figure 2:**
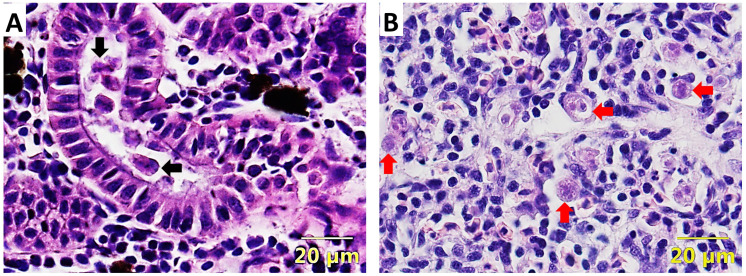
Histological sections of infected kidneys. **(A)** Brown trout kidney section shows intra-luminal sporogonic stages of *T. bryosalmonae* (arrows), **(B)** rainbow trout kidney section shows interstitial pre-sporogonic stages (red arrows) of *T. bryosalmonae* associated with macrophages (red arrows) (H&E staining).

### RNA-seq data analysis

The number of raw reads from each library ranged from 49.71 to 85.60 million. After quality filtering, 45.97 to 77.95 million reads were retained (**Supplementary Material 1**). Of these, 31.53–55.32 million reads, corresponding to 68.60–70.97% were successfully aligned to the genomes. The counts, fold changes and associated details for all transcripts from both fish species are provided in **Supplementary Materials 3**, **4**. DEGs were visualized in a MA (**Figure 3**) and a heatmap plot (**Figure 4**), which show differential gene expression patterns among transcripts. The statistical analysis (FDR adjusted *p*-value ≤ 0.01 and fold change ≥2 or ≤−2) revealed a total of 2,561 and 1,689 DEGs in infected kidneys of brown trout and rainbow trout, respectively.

**Figure 3:**
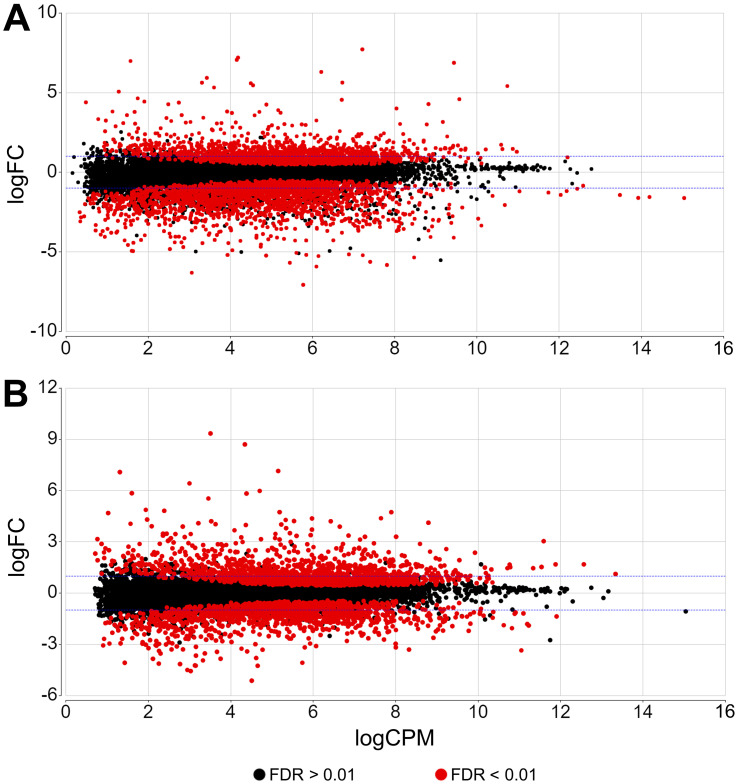
MA Plot for differential expression of brown trout **(A)** and rainbow trout **(B)**. X axis represents the average log transformed CPM (counts per million) expression values. Y axis represents the average log transformed fold changes of infected samples compared to controls. The dashed horizontal lines indicate the implemented fold change cut-offs. Genes significantly different (FDR < 0.01) between infected and control are colored in red.

**Figure 4:**
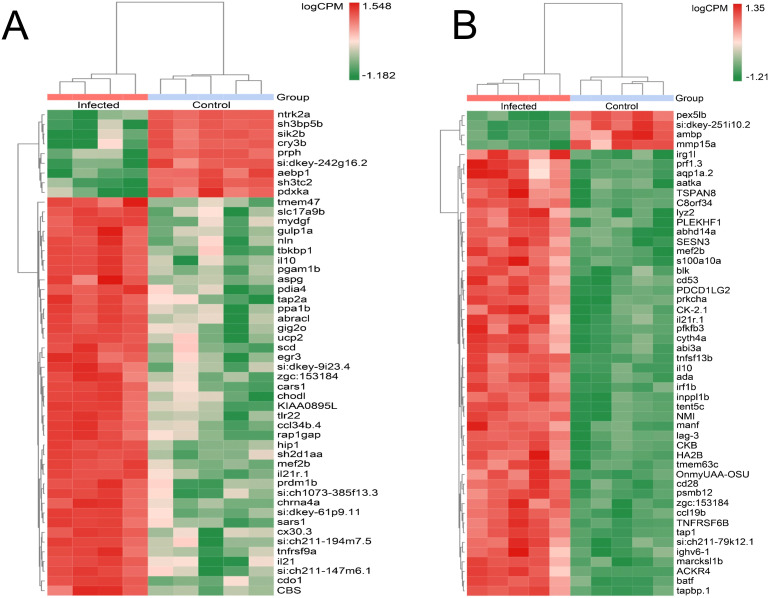
Heatmap showing the log transformed CPM (counts per million) expression values of the 50 most significantly regulated DEGs (ranked by FDR) for infected and control brown trout or rainbow trout samples. Red indicates high and green indicates low CPM values. Hierarchical clustering with the Euclidean distance formula was used to generate dendrograms on the left and top sides. **(A)** infected brown trout, **(B)** infected rainbow trout.

### Differential transcript analysis

A total of 880 and 1,681 transcripts were upregulated and downregulated in infected brown trout, respectively (**Supplementary Material 5**). In infected rainbow trout, a total of 782 and 907 transcripts were upregulated and downregulated, respectively (**Supplementary Material 6**).

A total of 240 upregulated and 317 downregulated transcripts were shared between infected brown trout and infected rainbow trout, respectively (**Supplementary Material 7**; **Figure 5**). Additionally, 4 shared transcripts were either upregulated in infected brown trout or downregulated in infected rainbow trout. Similarly, 16 shared transcripts were either upregulated in infected rainbow trout or downregulated in infected brown trout (**Table 1**).

**Figure 5:**
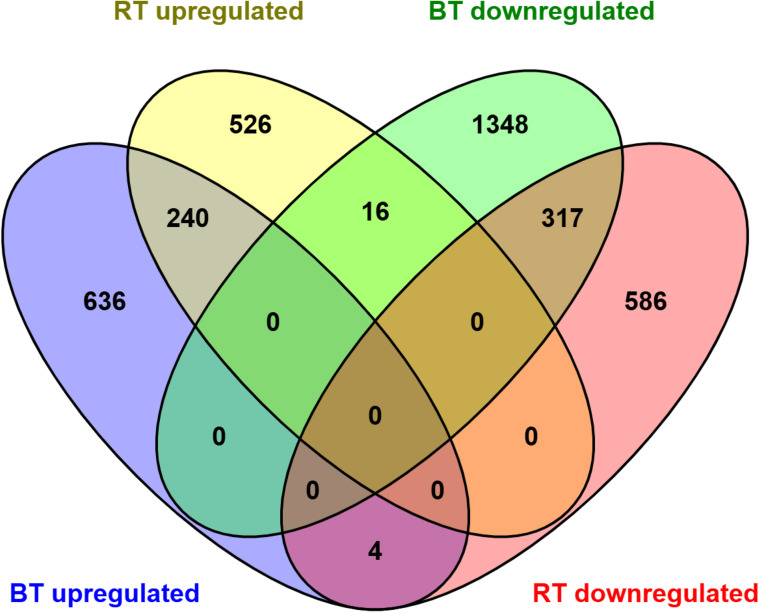
Venn diagram of total identified transcripts of infected brown trout and rainbow trout. Venn diagram represents the number of unique and shared genes between infected brown trout and rainbow trout. A total of 636 and 1,348 unique transcripts were upregulated and downregulated in the infected kidney of brown trout, respectively. In contrast, 526 and 586 unique transcripts were upregulated and downregulated in the infected kidney of rainbow trout, respectively. Four transcripts were shared between the upregulated genes of infected brown trout and the downregulated genes of infected rainbow trout. Similarly, 16 transcripts were shared between the upregulated genes of infected rainbow trout and the downregulated genes of infected brown trout.

**Table 1:** Infection regulated transcripts that exhibit opposite directional regulation in brown trout compared to rainbow trout.

Transcript	Function	Fold change	
		Infected brown trout	Infected rainbow trout
Upregulation in brown trout and downregulation in rainbow trout			
sil1	nucleotide exchange factor for the endoplasmic reticulum luminal chaperone HSPA5	2.24	-2.69
sez6b	neuronal development	3.67	-2.64
tafa5b	G protein-coupled receptor signaling pathway	2.15	-5.73
mafaa	transcription regulation	5.56	-2.41
Downregulation in brown trout and upregulation in rainbow trout			
slc24a4b	potassium-dependent sodium/calcium exchanger	-5.01	3.21
apoeb	apolipoprotein	-3.30	3.86
creb5a	cAMP response element and activates transcription	-3.19	2.64
itga5	transmembrane	-2.25	2.02
si:dkey-52j6.3	Ion transport	-2.31	2.56
slco4a1	Organic anion antiporter	-4.00	2.78
rergla	hydrolase	-3.25	5.64
ptges	prostaglandin E2 biosynthetic pathway	-2.05	2.06
PDE4B	cAMP phosphodiesterase activity	-2.27	2.10
B3GNT3	synthesis of poly-N-acetyllactosamine	-2.66	7.32
DDIT4L	negative regulator of the mTORC1 signaling pathway	-11.80	2.51
p4ha1b	collagen biosynthesis and structural integrity	-2.99	2.72
tert	telomere maintenance, tissue homeostasis, and organ regeneration	-4.35	2.01
hyal4	catabolism of glycosaminoglycans	-2.53	2.21
loxl2b	extracellular matrix remodeling	-4.39	2.10
dachd	organogenesis	-2.34	2.03

Four shared transcripts were either upregulated in infected brown trout or downregulated in infected rainbow trout. Similarly, 16 shared transcripts were either upregulated in infected rainbow trout or downregulated in infected brown trout.

A total of 636 and 1348 unique transcripts were upregulated and downregulated in infected brown trout, respectively (**Supplementary Material 8**). In contrast, 526 and 586 unique transcripts were upregulated and downregulated in infected rainbow trout, respectively (**Supplementary Material 9**).

### Gene ontology of uniquely expressed genes

The uniquely upregulated transcripts in infected brown trout were enriched in the cell cycle, cell cycle processes, meiotic cell cycle, and meiotic cell cycle processes. In infected rainbow trout, they were enriched in the regulation of biological quality, intracellular signal transduction, response to chemical stimuli, immune system processes, response to organic substances, cell migration, cell motility, and localization of cells.

The uniquely downregulated transcripts in infected brown trout were enriched in anatomical structure morphogenesis, cellular developmental processes, cell differentiation, animal organ development, and cell surface receptor signaling pathways. In infected rainbow trout, they were enriched in transmembrane transport, ion transport, nervous system development, anion transport, inorganic anion transport, and ammonium transmembrane transport.

The top 20 GO terms are presented in **Figure 6** and the details of enriched biological processes are provided in **Supplementary Material 9**.

**Figure 6:**
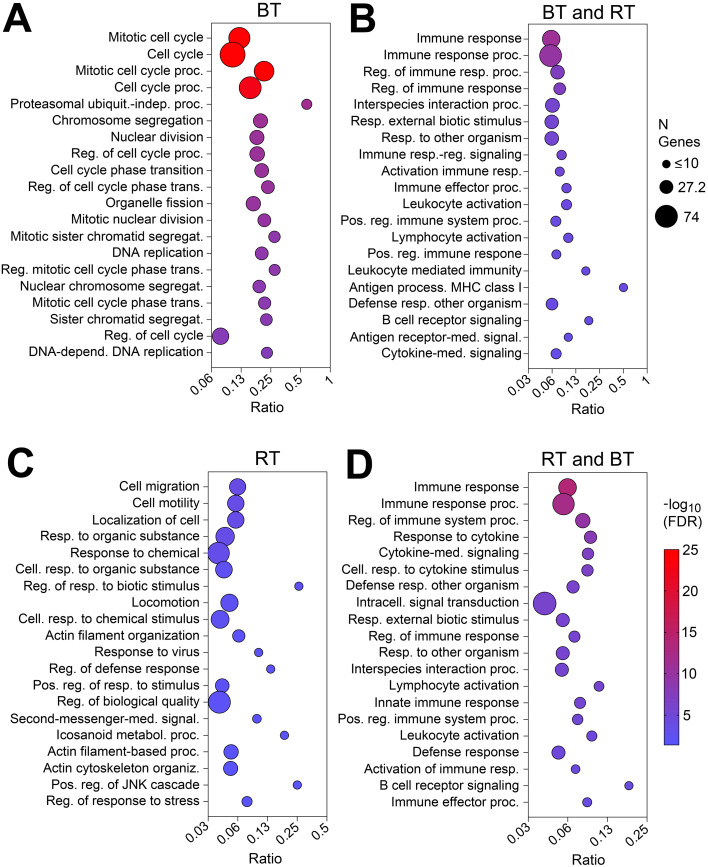
Bubble plot of enriched upregulated GO terms sorted by FDR *p*-value. Top 20 GO terms found uniquely in brown trout **(A)** compared to brown trout GO terms also detected in rainbow trout **(B)**. Top 20 GO terms found uniquely in rainbow trout **(C)** compared to rainbow trout GO terms also detected in brown trout **(D)**. Bubble size represents the numbers of DEGs from the data set associated with the GO terms. X axis represents the ratio of DEGs compared to the total number of genes associated with the respective GO terms. Colors indicate the FDR *p*-value.

### KEGG pathways of uniquely expressed transcripts

Cell cycle, cellular senescence, and proteasome pathways were enriched among uniquely upregulated transcripts in infected brown trout, whereas metabolic, focal adhesion, calcium signaling and MAPK signaling pathways were enriched among uniquely downregulated transcripts in infected brown trout.

Similarly, metabolic, MAPK signaling, neuroactive ligand-receptor interaction, and calcium signaling pathways were enriched among uniquely upregulated transcripts in infected rainbow trout, whereas only the metabolic pathway was enriched among uniquely downregulated transcripts in infected rainbow trout. The details of enriched KEGG pathways are presented in **Figure 7**; **Supplementary Material 10**.

**Figure 7:**
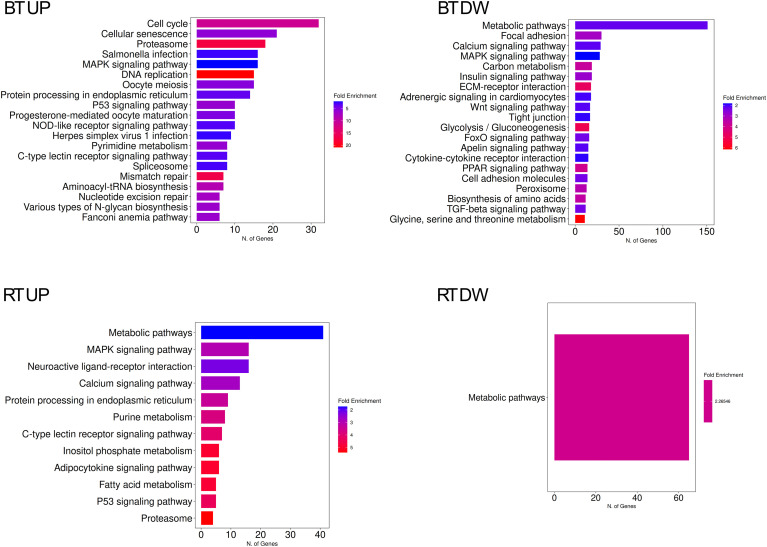
Enriched KEGG pathways detected for up- and down-regulated genes found in infected brown trout (BT) and rainbow trout (RT) samples. X axis represents the FDR p-values. Colors indicate the fold enrichment of the numbers of genes found in the data set compared to the total numbers of genes associated with the respective pathways.

### Validation of selected transcripts

The expression patterns of the six selected differentially regulated transcripts (APLNR2, CD22, CTSB, TSC22D3, TLR1, and SOCS3) as determined by qRT-PCR were consistent with the results of RNA-seq analysis (**Figure 8**). Both up- and downregulated transcripts showed comparable expression patterns in infected kidneys of brown trout and rainbow trout. Additionally, the magnitude and direction of expression changes obtained by qRT-PCR closely matched the fold-change derived from RNA-seq data. Linear regression analysis demonstrated a strong positive correlation between the two datasets (R² = 0.73, *P* < 0.05). This supports the reliability and consistency of the transcriptomic data. Overall, these findings validate the reliability of the RNA-seq results.

**Figure 8:**
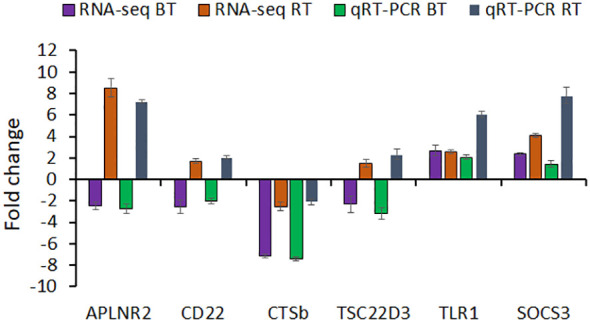
Validation of RNA-seq analysis by qRT-PCR on selected genes. The gene expression values are represented as relative fold change in the infected kidney compared to the un-exposed control kidney (n = 5). APLNR2: apelin receptor 2, CD22: B-cell receptor, CTSB: cathepsin-B, TSC22D3: TSC22 domain family protein 3, TLR1: toll-like receptor 1, and SOCS3: suppressor of cytokine signaling 3.

## Discussion

*T. bryosalmonae* exhibits different developmental outcomes in carrier (brown trout) and dead-end (rainbow trout) hosts. Previous studies have demonstrated that the parasite completes sporogenesis in brown trout but not in rainbow trout (Grabner and El-Matbouli, 2008; Okamura et al., 2011). Juvenile salmonids are recognized as the most susceptible vertebrate hosts to PKD, often exhibiting higher infection intensity and more pronounced disease manifestations (Schager et al., 2007; Mo et al., 2011; Dash and Vasemägi, 2014). Although adult fish can also acquire infection upon exposure (Feist and Longshaw, 2006; Lauringson et al., 2022), they generally show a lower propensity to develop overt disease, likely due to the development of partial protective immunity (Bailey et al., 2021). Clinical signs and pathological changes typically develop between 6- and 10-weeks post-exposure (wpe) in both brown trout and rainbow trout (Abd-Elfattah et al., 2014; Kumar et al., 2013; Schmidt-Posthaus et al., 2012), although disease progression may persist up to 12–17 wpe depending on host species, infection intensity and temperature (Shivam et al., 2021). Notably, individuals that recover from infection during the juvenile stage may persist as long-term carriers of *T. bryosalmonae*, thereby contributing to parasite maintenance in the environment. Such carrier fish can continue to release infective stages capable of transmitting the parasite to bryozoans for extended periods, with spore shedding reported to persist for several years, including up to five years in brown trout (Abd-Elfattah et al., 2014; Soliman et al., 2018). Consistent with these observations, the present study provides transcriptomic evidence indicating host-specific molecular environments that either promote or restrict sporogenesis of the European strain of *T. bryosalmonae* in the kidney tubules of infected juvenile fish.

### Carrier host-specific transcriptomic signatures

In infected brown trout, cell cycle regulation, DNA replication, and mitosis process genes such as *cdk1, ccnb1, ccna2, mcm2–7, pcna, aurka, plk1, e2f1* and *e2f3* were upregulated during PKD development, suggesting enhanced host cell proliferation within the kidney. These molecular responses are consistent with earlier findings of kidney hyperplasia and lymphoid proliferation during PKD (Clifton-Hadley et al., 1987; Bettge et al., 2009). In this context, enhanced proliferation of cells in the kidney reflects coordinated tissue remodelling associated with parasite development (Bailey et al., 2017). The upregulation of DNA repair and replication genes such as *rad51, brip1, chek1* and *pms2* further supports sustained cell division, which may be exploited by the parasite to complete sporogenesis. Conversely, cell adhesion, extracellular matrix and signaling genes such as *col1a1, itga6, egfr, notch1*, and *yap1* were downregulated in the infected kidney of brown trout, suggesting structural reorganisation and altered cell–cell interactions in the kidney in response to parasite development. These changes may facilitate parasite movement and sporogonic development within kidney tubules (Morris and Adams, 2008; Grabner and El-Matbouli, 2008).

Furthermore, the upregulation of proteasome components such as *psma, psmb*, and *psmd* and protein-folding and ER stress genes such as *hspa5, calr, and pdia3* in the infected kidney of brown trout, indicates higher protein turnover and activation of cellular stress response pathways. These biological processes are often associated with infection-induced stress and have been implicated in host–pathogen interactions. In these situations, parasites manipulate host proteostasis to enhance their survival (Kaushansky and Kappe, 2015; Hetz and Papa, 2018). The activation of hypoxia-inducible factor signaling, *hif1aa* and metabolic genes such as *fasn* and *dhodh* further suggests metabolic reprogramming creating conditions favorable for parasite persistence in infected fish tissues (Xie et al., 2021). Metabolic shifts toward biosynthesis and glycolysis pathways in infected tissues may support *T. bryosalmonae* development in kidney tubules.

From an immunological perspective, infected brown trout show activation of innate immune components, including *tlr22, tlr8, traf5, jak1, ripk2*, and *tnfa*. Toll-like receptor signaling is known to play a crucial role in pathogen recognition in fish (Sudhagar et al., 2020), and previous PKD studies have demonstrated modulation of innate immune responses during *T. bryosalmonae* infection (Gorgoglione et al., 2013; Bailey et al., 2019a; Sudhagar et al., 2019). The simultaneous upregulation of apoptosis regulators such as *casp7, casp8, tp53, birc5* and immune modulators suggests a balanced immune response in infected fish that may prevent excessive renal inflammation while permitting parasite persistence. Such controlled immune responses may help parasites survive without excessive host damage (Sitjà-Bobadilla, 2008; Sudhagar et al., 2019; Bailey et al., 2020).

### Dead-end host-specific transcriptomic signatures

In contrast, infected rainbow trout display a transcriptional profile dominated by activation of both adaptive and innate immune responses. T- and B-cell markers (*cd3, cd4, cd79a, cd79b, zap70, cd28, cd80, cd86*, and *ciita*) were upregulated in the infected kidney of rainbow trout, indicating activation of T- and B-cell mediated immunity during PKD. This is consistent with previous studies of lymphocyte proliferation and immune-mediated pathology in the infected kidney during PKD (Bailey et al., 2017). Previous transcriptomic and immunological studies have similarly reported upregulation of immune activation in rainbow trout during PKD, which is associated with clearance of parasites (Gorgoglione et al., 2013; Bailey et al., 2019a, 2020).

The induction of cytokine signaling genes like *il10, il11, il21r*, and *stat3* and inflammatory mediators such as *nfkb2, relb*, and *tnfaip3* suggests a regulated immune environment that balances activation and control of inflammation in infected rainbow trout (Secombes et al., 2011). The upregulation of regulatory molecules such as *socs* genes (*socs1* and *socs3)* suggests control of cytokine signaling to prevent immunopathology (Wang and Secombes, 2013; Kotob et al., 2018; Saleh et al., 2020). The upregulation of pattern recognition receptors and antimicrobial effector molecules such as *c3ar1, c5ar, lyz, mpeg1, and lbp/bpi* suggests effective pathogen recognition and elimination in the hosts (Whyte, 2007).

Infected rainbow trout also exhibited increased expression of cytotoxic and immune effector genes such as *prf1, runx3*, and *eomes*, indicating activation of cell-mediated cytotoxicity. These responses are known to play a key role in controlling intracellular pathogens (Zou and Secombes, 2016). Interestingly, infected rainbow trout also show increased expression of immune checkpoint genes such as *pdcd1lg2* and *lag3*. This suggests mechanisms at play to modulate excessive immune activation. However, despite these regulatory signals, the overall transcriptional profile indicates a strongly activated immune state. Furthermore, cell signaling, cytoskeletal organization, and immune cell migration genes like *cav1, iqgap1, rac2*, and *arpc family* are upregulated in the infected kidney of rainbow trout. These genes are essential for leukocyte trafficking and immune surveillance (Elks et al., 2011). However, this contrasts with brown trout, where cellular processes are more oriented toward proliferation and tissue remodelling.

Collectively, these findings suggest that rainbow trout mounts a strong and multifaceted immune response characterised by activation of adaptive immunity, cytotoxic pathways, and immune cell recruitment, accompanied by regulatory mechanisms that attempt to limit excessive inflammation. In contrast to brown trout, where coordinated proliferation and tissue remodelling predominate, the immune-dominated transcriptional profile in rainbow trout may lead to a microenvironment that restricts parasite development and prevents completion of sporogenesis.

### KEGG pathways of uniquely enriched transcripts

Our results demonstrate that brown trout as a carrier host, exhibit a pronounced upregulation of pathways associated with the cell cycle, cellular senescence and proteasome pathways, which likely support sporogenesis and the formation of intraluminal sporogonic stages in the kidney. These pathways may reflect cellular turnover and protein degradation processes in the fish that are often exploited by intracellular parasites to sustain their development (Glickman and Ciechanover, 2002; Ciechanover, 2005). In contrast, rainbow trout, functioning as a dead-end host show enhanced activation of processes related to the metabolic, MAPK signaling, neuroactive ligand-receptor interaction, and calcium signaling pathways reflecting a host response that may limit intraluminal sporogonic development. These pathways have been widely associated with host immune responses, stress signaling, and inflammatory regulation in fish during pathogen infections (Whyte, 2007; Wang and Secombes, 2013). The absence of sporogonic stages of the European strain of *T. bryosalmonae* in rainbow trout kidneys has been previously documented and linked to an ineffective parasite developmental environment, despite strong host immune activation (Grabner and El-Matbouli, 2008; Kumar et al., 2013).

Moreover, the downregulation of metabolic, focal adhesion and calcium signaling pathways suggests a host environment that supports parasite proliferation and development in the kidney of infected brown trout. This may be due to decreased cellular adhesion and changes in tissue integrity, which make it easier for the parasite to undergo sporogenesis. Similar downregulation of host structural and metabolic pathways has been observed in the kidney of infected fish, where *T. bryosalmonae* may manipulate host cellular processes to promote its development in kidney tubules and subsequent transmission to bryozoan hosts via urine (Kumar et al., 2013; Bailey et al., 2019a; Sudhagar et al., 2019). However, the downregulation of metabolic pathways in infected rainbow trout suggests disruption of physiological homeostasis associated with host defence responses and altered energy metabolism during infection (Fast et al., 2002). The differential regulation of metabolic and signaling pathways in the two fish species underscores distinct host-specific strategies in response to the parasite development.

### Shared transcripts between the carrier and the dead-end hosts

We found several genes including *sil1*, *sez6b*, *tafa5b*, and *mafaa* that were upregulated in infected brown trout but downregulated in infected rainbow trout. *sil1*, a nucleotide exchange factor, involved in endoplasmic reticulum stress regulation, may contribute to maintaining protein homeostasis in the fish during parasite development, ultimately supporting parasite survival in the carrier host (Zhao et al., 2005). Similarly, *sez6b* and *tafa5b*, associated with neuronal signaling and immune modulation, may be involved in host–parasite interaction and immune tolerance mechanisms. The upregulation of *mafaa* (5.56 fold), a transcription factor linked to cellular differentiation and metabolic regulation, further suggests that brown trout may create a permissive cellular environment that supports parasite sporogony in brown trout and suppresses it in rainbow trout.

On the other hand, a distinct set of genes, including *slc24a4b*, *apoeb*, *creb5a*, *itga5*, *slco4a1*, *rergla*, *ptges*, *PDE4B*, *B3GNT3*, *DDIT4L*, *p4ha1b*, *tert*, *hyal4*, *loxl2b*, and *dachd* were upregulated in the infected kidney of rainbow trout but downregulated in the infected kidney of brown trout. Most of these genes are associated with stress responses, inflammation, extracellular matrix remodeling, and cellular signaling pathways. For example, *ptges* (2.06 fold) is involved in prostaglandin synthesis and inflammatory responses, whereas, *PDE4B* (2.10 fold) regulates cyclic AMP signaling (Conti and Beavo, 2007). The upregulation of *itga5 (2.02 fold)*, *loxl2b (2.10 fold)*, and *p4ha1b* (2.72 fold) in infected rainbow trout suggests enhanced ECM remodeling and tissue repair processes that potentially reflecting a host attempt to counteract parasite invasion. Additionally, *DDIT4L* (2.51 fold) and *tert* (2.01 fold) are linked to cellular stress and survival pathways. This indicates that the activation of specific immune mechanisms may inhibit the development of sporogonic stages of the parasite in rainbow trout.

The differential regulation of solute carrier genes such as *slc24a4b* (3.21 fold) and *slco4a1* (2.78 fold) further suggests that alterations in ion transport and metabolic homeostasis occur in infected rainbow trout. Moreover, the upregulation of *apoeb* (3.86 fold) in infected rainbow trout suggests that lipid metabolism is involved in host defense mechanisms (Mahley and Rall, 2000). The differential transcriptome signatures in the infected kidneys of brown trout and rainbow trout support the idea that specific immune responses and their molecular pathways in each host affect the development of *T. bryosalmonae*. These findings align with previous studies on transmission, histopathological and transcriptomic patterns highlighting fundamental differences in both fish host immune responses (Soliman et al., 2018; Bailey et al., 2019a; Shivam et al., 2023), which support sporogenesis and intra-luminal sporogonic stages in brown trout but not in rainbow trout during PKD development.

### Limitations and future prospects

Although the present study provides comparative transcriptomic insights into host-specific development of *T. bryosalmonae* in brown trout and rainbow trout, certain limitations exist with the current study. Transcriptomic analyses were conducted at a single infection stage (10 wpe), which may not fully represent the temporal progression of host–parasite interactions during PKD. In addition, the study was performed under controlled laboratory conditions using juvenile fish, and therefore the observed molecular and pathological responses may differ under natural environmental conditions or in adult fish. Although parasite stages were confirmed histologically and immuno-histological prior to RNA sequencing, quantitative assessment of parasite burden using qPCR was not performed for individual fish. Furthermore, parasite-derived transcripts datasets were not analysed in the present study. Additionally, functional validation of differentially regulated host and parasite genes were beyond the scope of the current study. Differences in genome annotation resources and bioinformatic pipelines may also have influenced the number of genes available for downstream analyses. Finally, the study focused on a European strain of *T. bryosalmonae* and specific brown trout and rainbow trout stocks; therefore, the findings may not be directly transferable to other host populations or North American parasite lineages, which are known to exhibit distinct host–parasite interactions.

Future investigations integrating time-course transcriptomics, dual host–parasite RNA-seq, proteomics, and functional immune assays would improve understanding of the molecular mechanisms regulating parasite development, immune modulation, and sporogenesis in different salmonid hosts. Comparative studies involving European and North American parasite lineages, as well as juvenile and adult fish under field conditions, may further clarify host-specific adaptation and epidemiological dynamics of PKD.

## Conclusion

Our study provides comprehensive transcriptomic insights into the contrasting host–parasite interactions of the European strain of *T. bryosalmonae* in the carrier host, brown trout and the dead-end host, rainbow trout. Our findings reveal distinct molecular environments that underlie contrasting infection outcomes in the two species. Infected brown trout provide a permissive environment, which is characterized by enhanced cell proliferation, metabolic adaptation, and host-specific immune responses that facilitate parasite development, intraluminal sporogony and finally release of viable spores, which are infective for bryozoan hosts. In contrast, infected rainbow trout mount a strong and coordinated immune response that may restrict sporogenesis in the kidney tubules, preventing completion of its life cycle and resulting in a dead-end host. Additionally, this study expands our understanding of how molecular mechanisms determine host-specific immune responses during PKD and shows that the host regulates the development of the parasite and sporogenesis in kidney tubules by organizing different pathways that are involved in the regulation of these genes. Thus, this study enhances our understanding of fish–parasite interactions and establishes a molecular framework for exploring host tolerance and susceptibility in salmonids. These findings may provide an important basis for the identification of molecular markers associated with resistance or tolerance and developing future strategies to reduce the effects of PKD in farmed and wild salmonids. Future functional studies are needed to evaluate how the genes identified in this study function and to determine their potential application in the management of PKD.

## Data Availability

The datasets presented in this study can be found in online repositories. The names of the repository/repositories and accession number(s) can be found in the article/**Supplementary Material**.
